# Stress Induced Cardiomyopathy with Midventricular Ballooning: A Rare Variant

**DOI:** 10.1155/2015/154678

**Published:** 2015-06-04

**Authors:** Muhammad Umer Siddiqui, Michael C. Desiderio, Nicholas Ricculli, Arthur Rusovici

**Affiliations:** ^1^Department of Medicine, Englewood Hospital, 350 Engle Street, Englewood, NJ 07631, USA; ^2^Department of Cardiology, Morristown Medical Center, Morristown, NJ, USA

## Abstract

Stress cardiomyopathy (SCM) also referred to as the “broken heart syndrome” is a condition in which intense emotional or physical stress can cause fulminant and reversible cardiac muscle weakness. SCM most commonly involves the apical segment of left ventricle but newer and rare variants have recently been seen reported. We here report a case of rare midventricular variant of stress related cardiomyopathy. A 72-year-old female with past medical history, only significant for SVT, presented with an episode of severe substernal chest pain while hiking with her husband. She felt a significant heaviness in her chest and was short of breath. During her hospitalization she was found to have positive cardiac enzymes. EKG showed 1 mm downsloping ST segment changes. Ventriculogram during left heart catheterization revealed dyskinetic midventricle. Patient was diagnosed with midventricular SCM. The patient was placed on ACE inhibitor and beta-blocker and discharged in a well-compensated state. We suggest identifying these patients by standard lab testing, electrocardiography, echocardiography, and left heart coronary angiography and ventriculography. Management of this unique entity is similar to the other variants with close observation and treatment of accompanying heart failure, valvular dysfunction, and any arrhythmias that may develop.

## 1. **Introduction**

Stress cardiomyopathy (SCM) or left ventricular (LV) apical ballooning syndrome is characterized as a transient systolic ventricular dysfunction involving the apical or mid left ventricular segments, which is not associated with obstructive coronary artery disease [1]. Owing to the resemblance to an octopus trap, the Japanese initially referred to this syndrome as a “Takotsubo” cardiomyopathy [1]. Since that time, SCM has been identified throughout the globe. While the dyskinesis at the apical left ventricle is the archetypal example, other variations exist. Here we report a case of the midventricular variant of SCM.

## 2. **Case Presentation**

**Figure 1:**
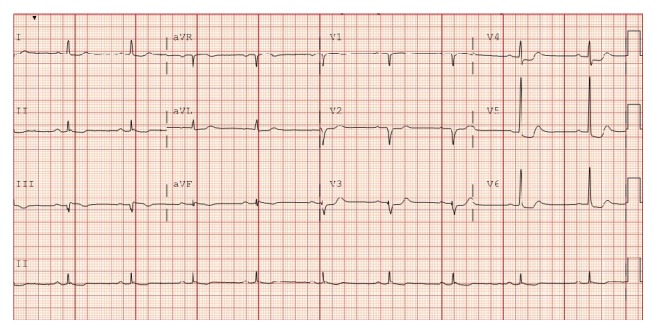
Presenting EKG of the patient with 1 mm ST segment depression in lateral chest leads.

**Figure 2:**
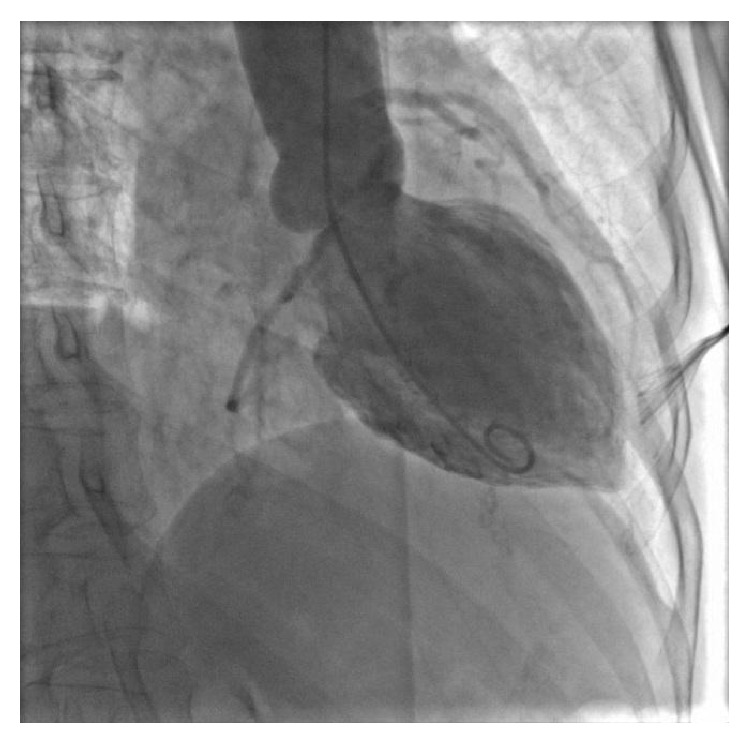
Cardiac catheterization showing left ventriculogram during diastole.

**Figure 3:**
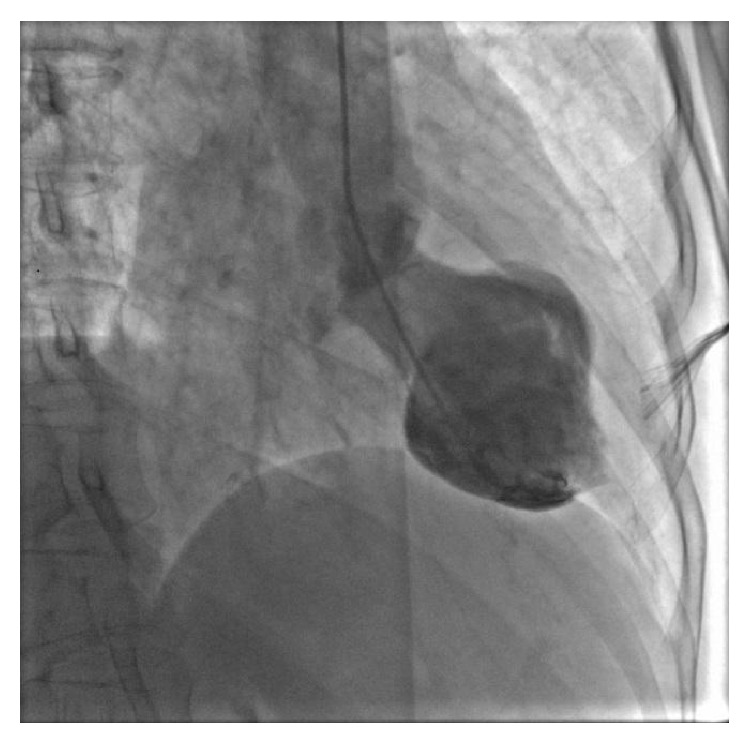
Cardiac catheterization showing left ventriculogram during systole. Midventricular ballooning with apical contraction.

A 72-year-old female with a history of supraventricular tachycardia, maintained on low dose aspirin and digoxin, presented to our institution with an episode of severe substernal chest pain that occurred while hiking with her husband. She described a significant heaviness in her chest associated with dyspnea and diaphoresis. After approximately 18 hours of nearly constant pain, the patient sought medical attention. Upon presentation to our hospital, cardiac examination was not significant for any valvular abnormality or signs of heart failure. Electrocardiogram (EKG) demonstrated normal sinus rhythm with 1.0 mm downsloping ST segment changes in lateral leads (Figure 1). She was found to have elevated cardiac enzymes with initial troponin at the level of 3.00 ng/mL and the next troponin level trended down to 2.16 ng/mL. Echocardiography identified midventricular systolic dysfunction with left ventricular ejection fraction (LVEF) of 40%. The patient subsequently underwent coronary angiography using standard technique, which revealed mild nonobstructive distal coronary artery disease. Left ventriculography using an angled pigtail catheter performed in the 30-degree RAO view (Figure 2) revealed abnormal ventricular function during systole. The basal and apical segments of the left ventricle were hyperdynamic, while the midventricular segments were akinetic (Figure 3). The patient was therefore diagnosed with a nonischemic cardiomyopathy, consistent with a midventricular variant of SCM. Hiking was thought to be the stressor causing SCM in her case. No other stressors were identified. The patient was placed on a beta-blocker, angiotensin converting enzyme (ACE) inhibitor, and an aldosterone inhibitor and was discharged home in a well-compensated state. Echocardiography performed 2 months following discharge revealed complete resolution of systolic dysfunction and normal left ventricular systolic function (EF 60%). Her aldosterone inhibitor was discontinued. Low dose beta-blocker and ACE inhibitor were continued. The patient remains asymptomatic at this time.

## 3. **Discussion**

Stress cardiomyopathy is believed to originate from extreme physical or emotional stress or result from acute medical illness [2–4]. Sharkey et al. identified 22 patients with SCM over a 32-month period and all of them had a psychological or emotional stress immediately preceding symptoms of SCM [4]. This suggests that the disorder may be caused by increased release of catecholamines leading to diffuse microvascular spasm and thereby causing myocardial stunning [5] or by direct catecholamine-associated myocardial toxicity [6]. Some studies have recognized a familial pattern; however, no genetic mutation has been identified [7, 8].

Patients who develop SCM most commonly present with acute substernal chest pain mimicking acute myocardial infarction [2, 4, 9] and, in severe cases, may develop acute valvular dysfunction such as mitral regurgitation, or dynamic left ventricular outflow tract obstruction, heart failure, or cardiogenic shock. The most common associated EKG finding is ST depression that is present in 34–56% of patients [10, 11]. The ventriculogram during angiography of the SCM patient typically demonstrates apical dyskinesis [2–5]. However, apical sparing variants have also been reported [12]. In one series of 256 patients, 82% were of the apical variety, 17% had midventricular dysfunction, and 1% were found to have basal dysfunction. Of note, 34% of cases demonstrated right ventricular involvement as identified by cardiovascular magnetic resonance imaging (CMRI) [13]. Despite the heterogeneity of ventricular dysfunction, the clinical characteristics of the variants are similar [14, 15]. Studies have shown that CMRI may be helpful in diagnosing SCM [3, 13]. CMRI is also helpful in differentiating SCM from myocarditis as myocarditis has patchy late gadolinium enhancement (LGE) on CMRI compared to absent LGE in SCM [13]. Our patient however did not get CMRI and decision was made to perform a cardiac catheterization to rule out coronary artery disease, causing wall motion abnormalities on echocardiogram.

Management of SCM is based upon managing left ventricular dysfunction [9]. Medical management includes beta-blockers, ACE inhibitors, and diuretics [9]. SCM is considered a transient disorder which resolves spontaneously and thus there is very limited data regarding duration of therapy. Some studies advise the use of aspirin for any coexisting atherosclerotic disease [9, 16]. In general, overall prognosis is good. In a study of 100 patients with SCM followed up for 4.4 years ± 4.6 months, the recurrence rate for SCM was 11.4% over 4 years after initial presentation and 31 patients continued to have episodes of chest pain. Seventeen patients died, but there was no difference in survival compared to an age- and gender-matched population [17].

The midventricular variant of SCM is being reported more frequently recently. We suggest identifying these patients by standard lab testing, electrocardiography, echocardiography, and left heart coronary angiography and ventriculography. Management of this unique entity is similar to the other variants with close observation and treatment of accompanying heart failure, valvular dysfunction, and any arrhythmias that may develop.

## 4. **Conclusion**

The diagnosis of stress cardiomyopathy should be entertained in postmenopausal women who present with typical angina symptoms, positive cardiac biomarkers, and EKG changes once obstructive coronary disease has been ruled out by angiography.

This case report highlights the atypical or midventricular variant of SCM. The clinical features including presentation, diagnostic imaging, treatment, and prognosis are similar among all variants. Wall motion abnormalities on echocardiography are not consistent with EKG changes and should raise the suspicion of SCM. Left ventriculography characteristically shows midventricular ballooning and coronary angiography may be normal or show mild-moderate nonobstructive coronary disease.

SCM represents a poorly understood clinical entity. Despite the variations of the ventricular dysfunction, management remains empirically treating the cardiomyopathy during this transient syndrome.
